# Monitoring live human mesenchymal stromal cell differentiation and subsequent selection using fluorescent RNA-based probes

**DOI:** 10.1038/srep26014

**Published:** 2016-05-20

**Authors:** Bojun Li, Ursula Menzel, Claudia Loebel, Hagen Schmal, Mauro Alini, Martin J. Stoddart

**Affiliations:** 1 AO Research Institute Davos, 7270, Davos Platz, Switzerland; 2 Department of Orthopedics and Traumatology, University Medical Center, Albert-Ludwigs University Freiburg, Germany; 3 Department of Orthopaedics and Traumatology, Odense University Hospital and Department of Clinical Research, University of Southern Denmark, Denmark

## Abstract

Investigating mesenchymal stromal cell differentiation requires time and multiple samples due to destructive endpoint assays. Osteogenesis of human bone marrow derived mesenchymal stromal cells (hBMSCs) has been widely studied for bone tissue engineering. Recent studies show that the osteogenic differentiation of hBMSCs can be assessed by quantifying the ratio of two important transcription factors (Runx2/Sox9). We demonstrate a method to observe mRNA expression of two genes in individual live cells using fluorescent probes specific for Runx2 and Sox9 mRNA. The changes of mRNA expression in cells can be observed in a non-destructive manner. In addition, the osteogenic hBMSCs can be prospectively identified and obtained based on the relative intracellular fluorescence of Sox9 in relation to Runx2 using fluorescence activated cell sorting. Relatively homogeneous cell populations with high osteogenic potential can be isolated from the original heterogeneous osteogenically induced hBMSCs within the first week of induction. This offers a more detailed analysis of the effectiveness of new therapeutics both at the individual cell level and the response of the population as a whole. By identifying and isolating differentiating cells at early time points, prospective analysis of differentiation is also possible, which will lead to a greater understanding of MSC differentiation.

Human bone marrow derived mesenchymal stromal cells (hBMSCs) have the potential to differentiate osteogenically, chondrogenically and adipogenically, and have been extensively studied for potential clinical therapies^1^. Osteogenesis of hBMSCs is of particular interest for bone tissue engineering. However, the lack of methods to reproducibly induce stable osteogenic differentiation severely limits their clinical use. In part this is due to the heterogeneous nature of the initial population, and the lack of methodologies to monitor cells at the individual, rather than the population level. The first problem is due to the lack of methods to isolate homogeneous hBMSCs. Current methods for isolation of hBMSCs either make use of the fact that the hBMSCs easily adhere to tissue culture plastic^2^, or are based on cell surface marker expression. Until now, significant research has been focused on CD marker-based attempts to isolate more homogeneous hBMSCs. For example, Stro-1, CD105, CD73 and CD90 have been used as positive markers to enrich hBMSCs^3^,^4^. Unfortunately no unique cell surface marker, or panel of surface markers, is presently known that is capable of isolating a pure population of hBMSCs. A recent study compared the CD marker profile of isolated MSCs to donor matched fibroblasts and could not detect any differences in CD marker tested^5^. This implies that hBMSCs as starting population for bone tissue engineering is heterogeneous, which in turn results in inherent inconsistency of the experimental outcomes.

A lack of methods to monitor hBMSC osteogenesis is another problem that hinders the clinical use of hBMSCs. Without a reliable method, it is difficult to accurately determine the effects of biomaterials and growth factors on hBMSCs *in vitro*. Hence it is no surprise there is difficulty in translating *in vitro* results to the *in vivo* situation. Typical methods for checking hBMSC osteogenesis include immunostaining of a number of osteogenic differentiation markers, and detection of the mRNA expression of these markers using RT-PCR. Compared to immunostaining, RT-PCR is more sensitive and provides quantitative information about mRNA expression in a population. However, there are two major drawbacks in RT-PCR: Firstly this method only shows the average mRNA expression, and it cannot easily detect mRNA expression in individual cells. Secondly, this method is destructive, and the cells cannot be reused for further tests. Hence there is a critical need for a new method to observe mRNA expressions in live cells and isolation of relative homogeneous stromal cells.

Master transcription factors, such as Sox9 (cartilage) and Runx2 (bone) are associated with cell differentiation pathways^6^,^7^. Our lab has previously demonstrated that the propensity of hBMSCs to differentiate osteogenically could be assessed by quantifying the ratio of Runx2/Sox9 mRNA message within the first week of osteogenic induction using RT-PCR^8^. While neither of these markers is specific, and the relative abundance varies from donor to donor, a ratio of the two has been shown to be predictive of phenotype *in vitro.* In order to observe mRNA expression of these two genes in live cells, a new method was developed using Smart-Flare^TM^ probes Runx2-Cy3 and Sox9-Cy5. Smart-Flare^TM^ probes is a nanoparticle-based system that can detect mRNA transcripts within living cells^9^. Gold nanoparticles are covalently labelled with capture oligonucleotides specific for Runx2 or Sox9 genes, and a fluorescently labelled short peptide. If complimentary mRNA is present, this peptide leaves the gold nanoparticle and begins to emit fluorescence. The system is available for two fluorochromes (Cy3 and Cy5), allowing for simultaneous detection of Runx2 and Sox9 mRNAs. Previous studies already report that this nanoparticle-based system can detect mRNA transcripts within living cells^10^,^11^. Here we have developed a fluorescence live monitoring system of hBMSCs to assess the ratio of Runx2/Sox9 in individual live cells. Furthermore, cells were isolated according to the relative intracellular fluorescence of Sox9 in relation to Runx2 at the single cell level using fluorescence activated cell sorting (FACS). Isolated cell populations were further investigated at the functional level by mineralization assay, identifying a distinct gated fraction with enhanced osteogenic differentiation potential and decreased proliferation rate. This method offers a more detailed analysis of the effectiveness of new therapeutics at the individual cell level, and a new way for isolating differentiating cells at early time points.

## Material and Methods

### Isolation and Expansion of Human BMSC

Human bone marrow was harvested from the iliac crest or vertebral body of eight patients, after full ethical approval and informed patient consent (Freiburg, EK-326/08) (mean age: 57.75 years; range: 24–83 years; male:female ratio: 5:3), to isolate hBMSCs using previously described protocols^12^,^13^. All methods were carried out in accordance with approved guidelines and all experimental protocols were approved by the Zürich Cantonal ethics committee. In brief, bone marrow was diluted 1:4 with PBS. The mixture was carefully layered on a Ficoll cushion (Histopaque-1077, Sigma), and centrifuged at 800×g for 20 mins. The mononuclear cells were collected from the liquid interface. A gated cell count (to exclude red blood cells) was performed using a cell Scepter 2.0 Automated Cell Counter (Millipore). Isolated mononuclear cells were seeded at a density of 50,000 cells/cm^2^ in cell culture flasks, and cultured in α-modified essential medium (α-MEM; GIBCO), 10% fetal bovine serum (Sera Plus, PAN-Biotec), 1% penicillin and streptomycin (GIBCO), and 5 ng/ml recombinant human basic fibroblast growth factor (bFGF). After 4 days, nonadherent hematopoietic cells were removed. Cells were incubated at 37 °C and 5% CO_2_ and the medium was refreshed every second day. Cell surface marker profiling was performed at passage 1 according to previous protocols^13^. The potency of the expanded attached cells to differentiate towards chondrogenic, osteogenic and adipogenic lineages was confirmed. Isolated hMSCs were expanded in monolayer with an initial seeding density of 3,000 cells/cm^2^ at 37 °C and 5% CO_2_, and received media changes every second day.

### Osteogenic differentiation of monolayer expanded cells

For induction of hBMSC osteogenic differentiation, passage 2 hBMSCs were seeded in 12 well plates with an initial seeding density of 10,000/cm^2^, and cultured in osteogenic induction medium for 6 days. The osteogenic induction medium (OM) contains Dulbecco´s modified Eagle’s medium low glucose (DMEM; GIBCO), 10% fetal bovine serum (Sera Plus, PAN-Biotec), 1% penicillin and streptomycin (GIBCO), 50 μg/ml ascorbic acid, 10 nM dexamethasone and 5 mM β-glycerol phosphate (Sigma-Aldrich). As negative control, cells were also cultured in growth medium (GM) which contains α -modified essential medium (α-MEM; GIBCO), 10% fetal bovine serum (Sera Plus, PAN-Biotec), 1% penicillin and streptomycin (GIBCO). The medium was changed every second day.

### Preparation of cells with SmartFlare^TM^ probes for flow cytometry

SmartFlare^TM^ probes Uptake-Cy3, Uptake-Cy5, Scramble-Cy3, Scramble-Cy5, Runx2-Cy3 and Sox9-Cy5 were kindly provided by Merk Millipore. These probes were reconstituted in 50 μl sterile nuclease free water (Milli-Q water), and stored at room temperature for use. hBMSCs were cultured in osteogenic induction medium or growth medium for 6 days, and SmartFlare^TM^ probes were directly added to the medium with a 1:1000 dilution. The dilution was chosen based on preliminary experiments (data not shown). After gentle shaking, cells were incubated with probes at 37 °C and 5% CO_2_ overnight (about 16 hours). Then cells were detached using 0.05% Trypsin-EDTA, and centrifuged at 2000 rpm for 10 mins. The cell pellets were resuspended in growth medium and incubated with 4′,6-Diamidino-2-phenylindole (DAPI, Sigma-Aldrich) at a dilution of 1:5000. Finally the cells were analyzed using flow cytometry.

### Flow cytometry and FACS

Flow cytometry analysis was performed using a BD Aria III machine, and data were analyzed using the BD FACS Diva 6.1.3 software. First dead cells which are positive for DAPI staining were excluded by gating for DAPI negative cells (Fig. S1A), and then the cell debris and clumps were excluded from the live cell population through a forward scatter (FSC)/ side scatter (SSC) gate (Fig. S1B). Doublets were excluded using FSC-A vs FSC-H gate (Fig. S1C). Finally the gated cells were analyzed, and cells in osteogenic induction medium are sorted into 4 populations (P1, P2, P3 and P4) based on the fluorescence intensity of Runx2-Cy3 and Sox9-Cy5. The sorted cells were collected in growth medium and reseeded in cell culture plates with a seeding density of 500/cm^2^. For RNA isolation, the sorted cells were collected in PBS with 1% FCS. Overlay histograms were made using the free flow cytometry data analysis software Cyflogic.

### Cell proliferation test

Sorted cells or unsorted cells grown in OM were seeded in a 96 well plate with an initial seeding density of 1,500 cells/cm^2^. After incubating in GM at 37 °C and 5% CO_2_ for 4 hours, most cells were attached. Unattached cells were washed away using GM for 3 times, and GM was changed every second day. On day 0 (cells attached and cultured on plate for 4 hours), day 3, day 6 and day 12, cells were detached using 0.05% Trypsin-EDTA (Gibco), and cell number was counted using Neubauer improved cell counting chamber. Each sample was repeated 3 times on each time point. The cell number was normalized to the cell number on day 0.

### Real-time quantitative PCR analysis

Total RNA was extracted using the Pure Link^TM^ RNA Micro Kit (Invitrogen) according to the manufacturer’s instructions. cDNA was synthesized using the SuperScript^®^ VILO cDNA Synthesis Kit (Invitrogen). RT-PCR was performed using the AB7500 Real-Time PCR System (Applied Biosystems) according to previously described methods^14^ Data analysis was performed using the ΔΔCT method, determined by normalization to RPL0 and cells grown in growth medium. Specific primer sequences used are listed below. For RT-PCR using assay on demand, the Applied Biosystems Reference No. is listed

RPL0:

Forward primer: 5′-TGG GCA AGA ACA CCA TGA TG-3′

Reverse primer: 5′-CGG ATA TGA GGC AGC AGT TTC-3′

Probe: 5′-AGG GCA CCT GGA AAA CAA CCC AGC-3′

Collagen I:

Forward primer: 5′-CCC TGG AAA GAA TGG AGA TGA T-3′

Reverse primer: 5′-ACT GAA ACC TCT GTG TCC CTT CA-3′

Probe: 5′-CGG GCA ATC CTC GAG CAC CCT-3′

hOC:

Forward primer: 5′-AAG AGA CCC AGG CGC TAC CT-3′

Reverse primer: 5′-AAC TCG TCA CAG TCC GGA TTG-3′

Probe: 5′-ATG GCT GGG AGC CCC AGT CCC-3′

hRunx2

Forward primer: 5′-AGC AAG GTT CAA CGA TCT GAG AT-3′

Reverse primer: 5′-TTT GTG AAG ACG GTT ATG GTC AA-3′

Probe: 5′-TGA AAC TCT TGC CTC GTC CAC TCC G-3′

hSox9

Applied Biosystems Reference No. Hs00165814_m1.

hALP

Applied Biosystems Reference No. Hs00758162_m1.

### Alizarin red staining

3.4226 g Alizarin Red S (Sigma-Aldrich A5533) was dissolved in 150 ml Milli-Q water, and the pH was adjusted to 4.1–4.3. Sorted or unsorted cells were cultured in osteogenic induction medium or growth medium for 3 weeks. Cells were fixed with 4% formaldehyde and incubated in Alizarin Red solution at room temperature for 1 hour on a rotating plate. The sample was washed with Milli-Q water for 10 times, and visualized using an inverted microscope (Axiovert 40 CFL,Zeiss).

### Statistics

Data presented for mRNA expression are given as mean ± standard deviation of the mean of pooled data from multiple experiments, using individual donors. All mRNA expression values were normalized to the values of unsorted cells in growth medium. Statistical analysis was performed using SPSS 16.0 statistical software (SPSS, Inc., Chicago, IL). Differences between groups were tested for statistical significance by means of a Kruskall-Wallis one-way analysis of variance test. A difference of p < 0.05 was considered significant.

### Detection of Runx2 and Sox9 gene expression in live cells using single fluorescence probes

**Figure 1:**
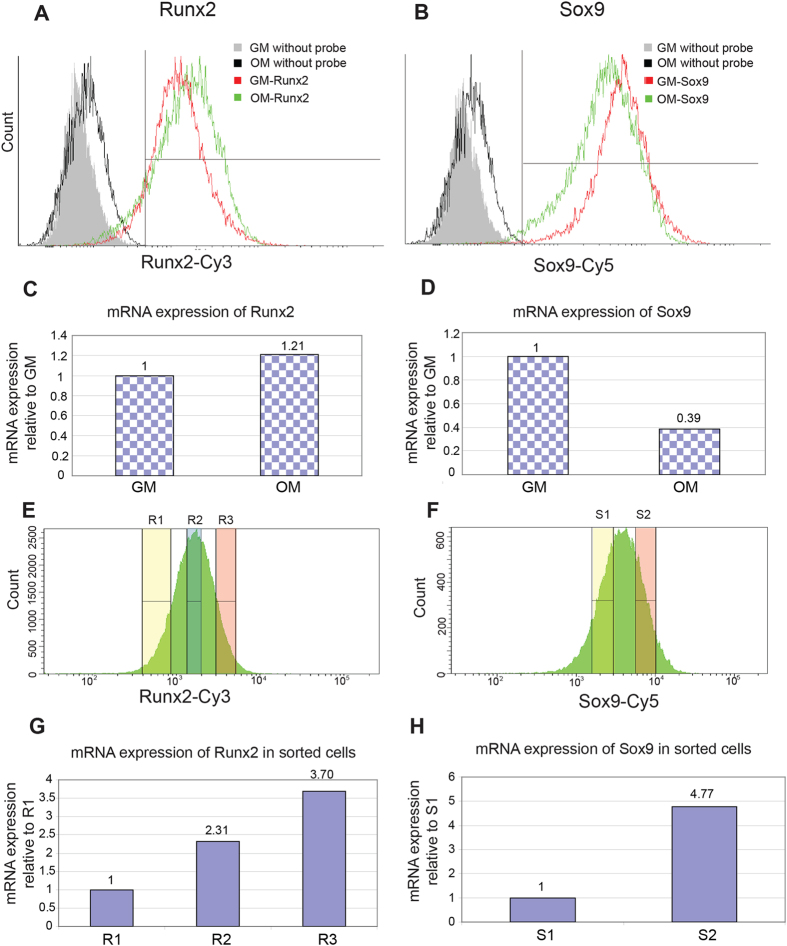
SmartFlare^TM^ probes could detect mRNA expression in live cells. (**A,B**): Histogram of fluorescence intensity of hBMSCs with single Runx2-Cy3 (**A**) or Sox9-Cy5 (**B**) probes in growth medium (GM, red line) or osteogenic medium (OM, green line) after 6 days culture. As negative control, the histogram of fluorescence intensity of hBMSCs in GM or OM without probes was also shown. (**C,D**) RT-PCR results of Runx2 (**C**) and Sox9 (**D**) expressions in hBMSCs in GM or OM after 6 days culture. (**E**) Cells in OM were sorted based on the intensity of Runx2-Cy3, R1: Low intensity, R2: Medium intensity, R3: High intensity. (**F**): Cells in OM were sorted based on the intensity of Sox9-Cy5, S1: Low intensity, S2: High intensity. (**G,H**) RT-PCR results of Runx2 (**G**) and Sox9 (**H**) expressions in sorted cells.

Relative fluorescence intensity in individual live cells can be observed after addition of the mRNA specific probe (Fig. 1). Compared to cells without SmartFlare^TM^ probes, 87% cells in growth medium (GM) and 81% cells in osteogenic medium (OM) took up the Runx2-Cy3 probe and produced a fluorescence signal (Fig. 1A). 97% cells in GM and 95% cells in OM took up Sox9-Cy5 probe and showed the fluorescence signals (Fig. 1B). As positive control, more than 90% cells took up the uptake-Cy3 or uptake-Cy5 probes in GM or DM (data not shown). It is clear that most cells could take up the probes, and the fluorescence signals are strong enough to be detected by flow cytometry. A shift in relative mean fluorescence intensity (mfi), indicating a change in mRNA levels, was observed between cells in OM (green line) and GM (red line) in Fig. 1A,B. For Runx2 expression, an increase in mRNA levels after osteogenic induction was observed with the mfi value changed from 1,324 to 1,810 (1.37 times). In contrast, for Sox9 mRNA expression a decrease in mRNA levels was observed after osteogenic induction The mfi decreased from 6,269 to 3,300 (0.53 times). The average Runx2/Sox9 ratio from cells cultured in growth medium or osteogenic medium changed from 0.211 to 0.548 (2.6 times).

To correlate mRNA expression levels measured by relative mfi values to quantitative mRNA values, RT-PCR analysis was performed. The cells in GM or OM without fluorescence probes and labeled sorted cells were collected, and their mRNA message quantified by RT-PCR.

Runx2 mRNA expression levels measured by RT-PCR increased slightly after osteogenic induction to 1.21, whereas expression levels for Sox9 mRNA decreased to a large extent to 0.39 relative to mRNA levels from cells cultured in GM (Fig. 1C,D). These changes in expression levels result in a Runx2/Sox9 ratio of 3.13 in OM compared to GM. This is consistent with a previous report that a higher Runx2/Sox9 ratio indicates hBMSC osteogenic differentiation^8^. Results obtained by RT-PCR correlated with results from flow cytometry and show that the fluorescent probes can provide semi-quantitative information about mRNA expression in a cell population.

Theoretically mfi levels of fluorescent RNA probes correspond to mRNA expression levels in individual live cells. In order to confirm this, cells which cultured in OM for 6 days and tagged with probes specific either for Runx2 or Sox9 were separated by FACS in populations with different mfi levels (Fig. 1E,F) and mRNA levels of sorted cells were quantitatively assessed using RT-PCR. Cells with high fluorescence intensity (R3, mfi 3,358) show higher Runx2 mRNA expression levels than cells with low fluorescence intensity (R1 mfi 603), while cells with medium fluorescence (R2, mfi 3,358) show medium Runx2 expression. (Fig. 1E,G). Corresponding results were obtained for cells sorted on different mfi levels for Sox9-Cy5 (Fig. 1F,H).

### Runx2 and Sox9 fluorescent probes can detect mRNA expression of two genes in individual live cells simultaneously and be used for cell sorting

Runx2 is considered as a positive regulator and biomarker for hBMSC osteogenic differentiation^15^. Further study showed that the ratio of Runx2/Sox9 is more accurate at predicting the hBMSC osteogenic differentiation at early time points^8^. Therefore, monitoring the expression of both genes, Runx2 and Sox9, simultaneously is necessary to predict the osteogenic potential of hBMSC. Probes for Runx2 and Sox9 were added simultaneously to GM or DM on day 6, and the mean fluorescence intensities were quantified on day 7 using flow cytometry. Live/dead staining with DAPI was included to eliminate background fluoresence from cell debris and dead cells. A summary of the gating strategy applied for flow cytometry analysis is shown in Fig. S1.

To determine whether cells could uptake both probes and show fluorescence signals, cells incubated with dual both probes were compared with unstained controls. For uptake control, more than 90% cells took up the uptake-Cy3 or uptake-Cy5 probes in GM or DM (data not shown). For Runx2 specific probes, 80% cells in GM and 83% cells in OM show fluorescence signal (Fig. S2A). For Sox9, 97% cells in GM and 90% cells in OM show fluorescence signal (Fig. S2B). It suggests that most cells could take up both Runx2 and Sox9 probes, and show detectable signal.

**Figure 2:**
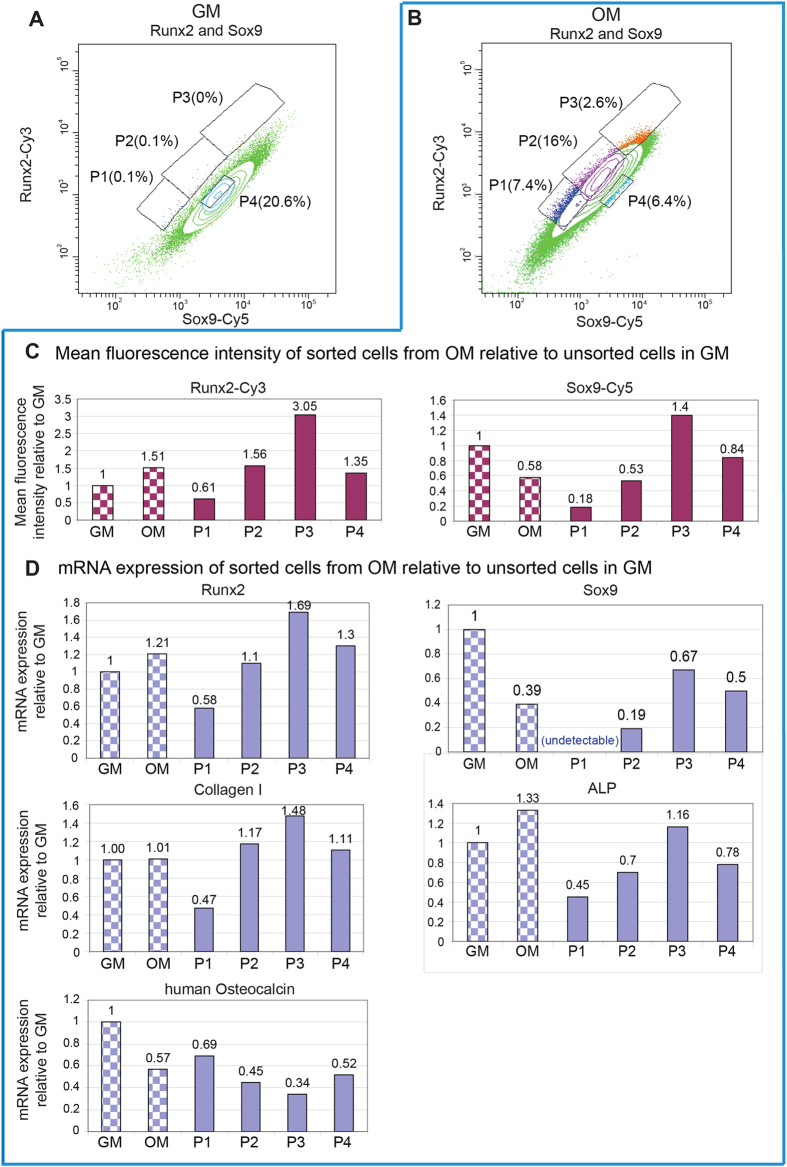
Simultaneous detection of Runx2 and Sox9 mRNA expression in individual live cells using two specific SmartFlare probes. (**A,B**) hBMSCs incubated with two SmartFlare probes Runx2-Cy3 and Sox9-Cy5 in GM (**A**) or OM (**B**) after cultured for 6 days. The fluorescence intensity was checked using flow cytometry on day 7. Cells in OM were further gated into 4 groups (P1–P4) based on the relative intracellular fluorescence of Sox9 and Runx2. P1 (low Sox9/ low Runx2), P2 (med Sox9/ med Runx2), P3 (high Sox9/ high Runx2), P4(high Sox9/ med Runx2). (**C**): Mean fluorescence intensity (mfi) of sorted cells (P1–P4), and unsorted cells in GM or OM (dashed column). The mfi of sorted cells are normalized to unsorted cells in GM. (**D**) Real time PCR results of Runx2, Sox9, Collagen I, ALP and human Osteocalcin expressions from sorted osteogenic induced hBMSCs or from unsorted cells in GM or OM (dashed column). mRNA expressions are normalized to unsorted cells in GM.

mRNA expression of two genes can be simultaneously detected in live cells (Fig. 2A,B). A clear shift in profile was observed between cells in GM and OM. The whole population of cells in OM shifts to the left (Fig. 2B), which shows a decrease in Sox9-Cy5 fluorescence. The mfi of Sox9-Cy5 is 4,537 in GM, and decreased to 2,641 in OM. The mfi of Runx2-Cy3 is 1,008 in growth medium, and increased to 1,521 in osteogenic induction medium. Both the value of mfi (numeric presentation of result) and the population shift (graphic presentation of the same result) show the similar expression pattern of Runx2 and Sox9 as seen in Fig. 1 using a single probe and RT-PCR. This experiment was repeated with 8 different donors. Similar results were observed for all samples, and showed that hBMSCs could take up both probes, and show mRNA expression of two different genes can be detected simultaneously. Compared with RT-PCR, this method is sensitive enough to observe changes of mRNA expression in individual hBMSCs at an early time point after osteogenic induction.

To determine whether the observed shift in Fig. 2B is really due to the osteogenic differentiation of hBMSCs, cells in OM were sorted into 4 groups based on the fluorescence intensity of Sox9-Cy5 and Runx2-Cy3. The sorting gates were as follows: P1 (low Sox9/ low Runx2), P2 (medium Sox9/ medium Runx2), P3 (high Sox9/ high Runx2) and P4 (high Sox9/ medium Runx2). The mfi of sorted cells were normalized to unsorted cells in GM, and the results clearly showed the changes of mfi of sorted cells (Fig. 2C). Gates P1, P2 and P3 were chosen as the number of events in these populations is below 1% in GM, but increases when cells are cultured in OM. For example, only 0.1% of cells are in region P1 in GM, however in OM this population increased to 7.4%. For region P4 (high Sox9/ medium Runx2), the percentage of cells decreases from 20.6% in GM to 6.4% in OM.

**Figure 3:**
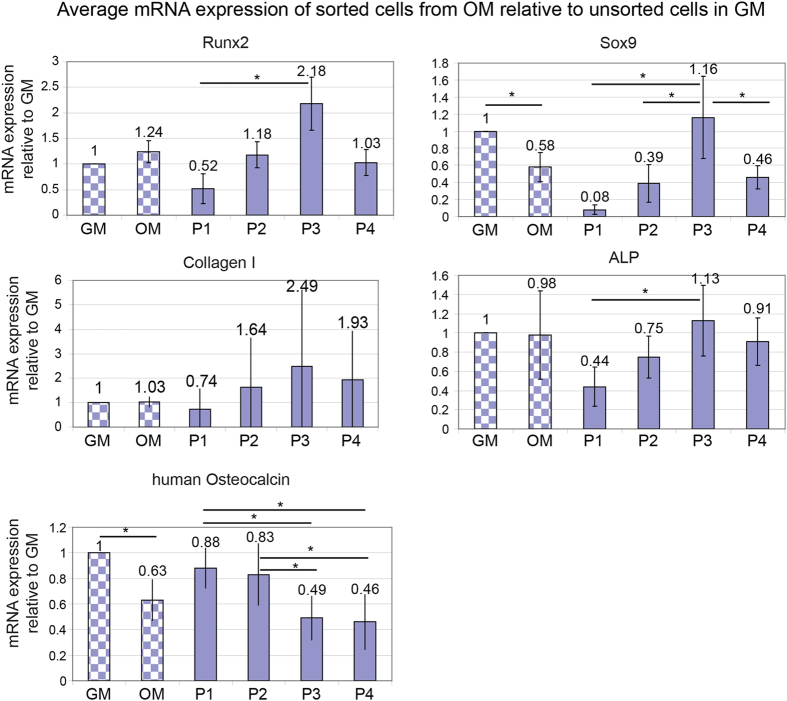
Average mRNA expression of Runx2, Sox9, Collagen I, ALP and human Osteocalcin from sorted osteogenic induced hBMSCs P1–P4, and unsorted cells in GM or DM (dashed column). mRNA expressions are normalized to unsorted cells in GM. Values represent the mean ± standard deviation (n = 5), *p < 0.05.

The mRNA expression of sorted cells from OM and unsorted cells in GM or OM were also analyzed using RT-PCR. The mRNA expression for Runx2 and Sox9 is consistent with the flow cytometry results (Fig. 2D). Quantitative mRNA analysis by RT-PCR for P1 shows low Runx2 and low Sox9 expression. In this sample, Sox9 expression is too low to be detected, and Runx2 is only 0.58 compared to cells in GM. P2 shows higher Runx2 and Sox9 expression than P1, but lower than P3. The RT-PCR results perfectly matched the flow cytometry data in Fig. 2C. The RT-PCR analysis was repeated using 5 different donors. The average RT-PCR results are shown in Fig. 3. It is clear that the RT-PCR results correspond with flow cytometry results using the fluorescent probes, and shows that P1 has the lowest Sox9 and Runx2 expression.

Interestingly, according to average mRNA expression of Runx2 and Sox9 using RT-PCR, P1 shows the highest Runx2/Sox9 ratio (6.75), and P2 show medium ratio (3.9). P3 (2.0) and P4 (2.3) show the lowest ratio (Fig. 3). According to the previous report that the ratio of Runx2/Sox9 is more accurate to predict hBMSC osteogenic differentiation, the different Runx2/Sox9 ratio of P1–P4 indicates different differentiation potential of the sorted cells^8^.

To further investigate the differentiation of sorted cells, mRNA expression of the early osteogenic marker alkaline phosphate (ALP), Collagen I, and late osteogenic differentiation marker human osteocalcin (OC) was determined. As shown in Fig. 2D, P1 has the lowest ALP and collagen I expression. P2 has medium expression of ALP and collagen I. While P3 has the highest ALP and collagen I. Runx2, ALP and collagen I are generally considered as early markers for osteogenic differentiation^16^,^17^. These 3 early genes show a similar trend. It suggests that P1 has the lowest early osteogenic marker expression. This result is repeated using 5 different donors including the donor showed in Fig. 2. The other results of 4 additional donors are shown in Fig. S3. The average RT-PCR results confirm the trend (Fig. 3). In contrast to early osteogenic markers, P1 shows the highest OC expression, and P3 and P4 shows the lowest OC expression (Fig. 2D and Fig. S3). The trend was similar among all 5 different donors. Unlike Runx2, ALP and collagen I, OC is considered as a late marker for osteogenic differentiation^18^. This result suggests that P1, P2 and P3 are at different stages of osteogenic differentiation, and it seems that P1 is at relatively late stage, and P2 and P3 are at earlier stages.

### Sorted cells based on two fluorescence probes show different proliferation rate and osteogenic differentiation potential

**Figure 4:**
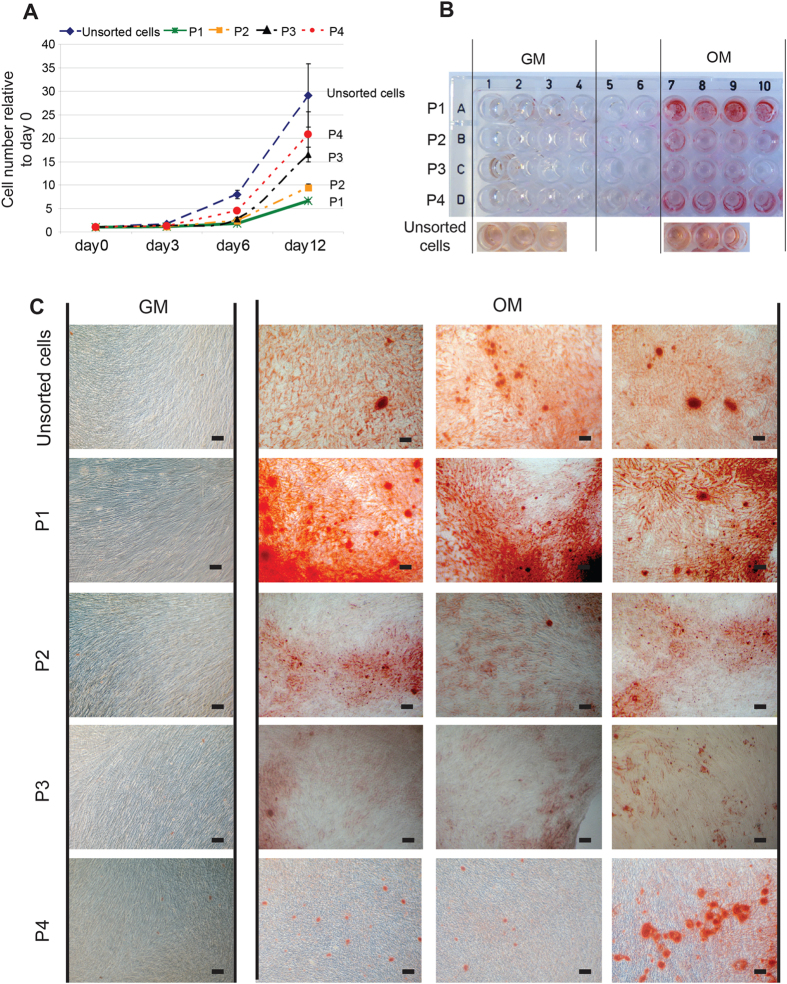
Sorted cells based on SmartFlare probes Runx2-Cy3/Sox9-Cy5 show different proliferation rate and osteogenic differentiation potential. (**A**) The sorted cells from OM (P1: low Sox9/low Runx2, P2: med Sox9/ med Runx2, P3: high Sox9/ high Runx2, P4: high Sox9/ med Runx2) and unsorted cells that were incubated in OM for 6 days were seeded and cultured further in GM. Cell number was counted on day 0, day 3, day 6 and day 12, and the cell number was normalized to unsorted cells. (**B**) Alizarin red staining of sorted cells after 3 week culture in GM or OM. (**C**) Microscope view of Alizarin red staining of sorted cells in (**B**). The pictures shown here were random taken in different wells. Scale bar = 100 μm.

To further characterize the sorted cells, the cells were reseeded on tissue culture plastic, and cultured in GM. Cell number was quantified on day 0, day 3, day 6 and day 12. Cells grown in OM, which were not sorted using FACS and SmartFlare^TM^ probes (unsorted cells), have the highest proliferation rate. After culture for 12 days, the cell number increased about 30 times compared to day 0. P2, P3 and P4 have a medium proliferate rate. P1 has the lowest proliferation rate, and cell numbers increased only approximately 6 fold after culture for 12 days (Fig. 4A).

Alizarin red staining was performed to test the calcium deposition of sorted cells in GM or OM after 3 weeks. In GM, no staining was observed in any of the groups, even though the sorted cells were incubated with OM for 6 days before sorting (Fig. 4B,C). After culture for 3 weeks in OM, P1 showed the highest alizarin red staining among all sorted cells (P1–P4), and unsorted controls (Fig. 4B,C). P2 and P3 show weaker staining than P1 and unsorted cells, but stronger than P4. P4 shows the weakest staining. However, osteogenic differentiation of P4 is not quite as consistent as the other groups. In some areas of the wells, cells show relatively stronger staining than other areas (Fig. 4C lower right picture). This indicates that the P4 population is not homogeneous, and some cells in P4 have a higher osteogenic differentiation potential than other cells in P4. This experiment was repeated 3 times using 3 different donors, and all showed similar results (Fig. 4 and Fig. S4). It confirms that osteogenically induced hBMSCs are not homogeneous, and cells have different proliferation rates and osteogenic differentiation potential. These heterogeneous cells can be sorted into a relatively homogeneous population based on the mRNA expression of Runx2 and Sox9. The sorted cells are relatively homogeneous for osteogenic differentiation in P1, P2 and P3. Among these sorted cells, P1 has the highest osteogenic differentiation potential and lowest proliferation rate, a phenotype more associated with osteoblasts.

## Discussion

Due to the lack of methodologies to monitor hBMSC activities and isolate a relatively homogeneous hBMSCs, the clinical use of hBMSCs is still limited. There is a critical need for development of a new method to observe stem cell activities in live cells. In our study, a novel method is developed using two Smart-Flare^TM^ probes Runx2-Cy3 and Sox9-Cy5. This new method is relatively easy to perform and the workflow for cell sorting is comparable to that used for CD marker sorting. The two probes can be directly added into the medium and after overnight incubation the mRNA expression level can then be visualized via microscopy. Additionally, sorting or quantification by FACS is also possible, providing a novel way to observe mRNA expression in live cells and isolate cells based on ther relative expression.

Smart-Flare^TM^ is a new live cell RNA detection technology based on gold nanoparticles conjugated to oligonucleotides duplexed with reporter strands. The particles are endocytosed by live cells using the cells’ own endocytosis machinery. Over time, the probes exit cells without adverse effects. In previous studies, single probes were used to detect gene expression and sort cells^10^. In our study, most cells (more than 80%) took up Runx2 or Sox9 specific probes and showed a fluorescent signal. This uptake ratio is higher than a previous report using murine embryonic stem cells^10^. Cell sorting data further confirmed that the intensity of fluorophore represents the mRNA expression in live cells (Fig. 1). By applying both probes at the same time, simultaneously quantifying two genes in live cells can be achieved (Fig. 2). A major advantage of this is that each cell acts as its own control for uptake and dissociation of probe, greatly enhancing the data obtained compared to the averages obtained when the controls are in separate wells. Previous studies have used live mRNA analysis for cell sorting, however these studies used individual probes^10^. In many cases, single gene expression cannot actually predict cell activities, and it limits the usage of this method. We observed that under high density monolayer conditions the decrease in Sox9 alone was not sufficient to predict the osteogenic differentiation potential. However, the Sox9/Runx2 ratio was able to detect osteogenesis under identical conditions (data not shown) demonstrating that monitoring expression of two genes provides a more sensitive analysis than individual genes alone.

Runx2 is expressed in both undifferentiated and osteoblast differentiated cells, as is Sox9. Thus each marker alone may not be an optimal marker for subtyping hBMSC populations. However, as one predominates over the other, the ratio becomes a more accurate predictive marker. Combination of the mRNA expression of Runx2 and Sox9 provides a more accurate prediction of osteogenic potential of hBMSC^8^. In general, these two transcription factors can only be detected via destructive methods, either intracellular immunostaining or PCR. A novel method is developed here to detect mRNA expression of two genes within live cells simultaneously. As shown in Fig. 2, the mRNA expression of Runx2 and Sox9 could be measured using flow cytometry, and the changes of mRNA expression caused by osteogenic induction for 6 days are clearly observed. Unlike previous methods, cells are still alive after the assay is performed. This provides an opportunity to sort cells for further testing. Osteogenically induced hBMSCs were sorted into 4 groups (P1–P4) based on the fluorescence intensity of Sox9-Cy5 and Runx2-Cy3. P1 (low Sox9/ low Runx2), P2 (medium Sox9/ medium Runx2), P3 (high Sox9/ high Runx2) and P4 (high Sox9/ medium Runx2). RT-PCR results of Runx2 or Sox9 expression of sorted cells match the results from flow cytometry (Fig. 2C,D), demonstrating the mRNA expression observed using two fluorescent probes is consistent with standard RT-PCR results.

Further analysis of sorted cells shows that P1, P2 and P3 have different gene expression patterns, though they mainly appeared in osteogenic induced cells (Fig. 2B). P1 has low early osteogenic marker expression, such as Runx2, ALP and collagen I, but high late osteogenic marker (OC). On the contrary, P3 has high early osteogenic marker expression, but low late osteogenic marker expression. P2 has a medium expression pattern. Though statistical analysis of ALP, collagen I and OC of 5 different donors did not show a statistically significant difference between P1 and P2, the trend is similar in all 5 donors (Fig. 2 and Fig. S3). The lack of significance suggests that the gate strategy needs to be further refined to take donor variation into account. It has been reported that the whole process from an undifferentiated hBMSC to an osteoblast occurs along a distinct pathway, and each of the stages was characterized by a particular gene expression pattern^6^. As an important transcription factor, Runx2 has been reported to promote osteogenic differentiation at early stages, but inhibit osteoblast maturation at a later stage^6^,^19^. P1 has low Runx2, low ALP and high osteocalcin expression which indicate P1 is at a relatively late stage of osteogenic differentiation. P2 and P3 are at an early to intermediate stage of osteogenic differentiation. This shows it is possible to isolate cells based on actual differentiation state, rather than time after application of stimulus. We propose that the high levels of most markers seen over multiple time points during standard osteogenic assays is due to the mixed populations present at different stages of differentiation, e.g. P1 providing high OC while P3 is providing high ALP and COL1.

The sorted cells grown in OM for 6 days were reseeded and cultured in GM or OM. Cell proliferation and osteogenic differentiation was determined using cell counting and alizarin red staining respectively. It shows that P1 has the lowest proliferation rate, but highest osteogenic differentiation potential. P2 and P3 have higher proliferation rate than P3 but lower osteogenic differentiation potential. P4 has the highest proliferation rate among sorted cells, but lowest osteogenic differentiation potential. It has been reported that hBMSCs with high proliferation rate have less osteogenic differentiation potential^20^. This is consistent with our finding. Taken together, our results suggest that we successfully isolated a relatively homogeneous cell population (P1) with a low proliferative but high osteogenic potential. Though RT-PCR results indicates that P1 is at relative late stage of osteogenic differentiation after osteogenic induction for 6 days, Alizarin red staining shows that no clear calcium deposition was found in P1 cells after cultured in GM for 3 weeks. It suggests that P1 cells did not fully differentiate into mature osteoblasts on day 7, and continued osteogenic induction is needed to induce calcium deposition. However, it should be noted that primary human osteoblasts also do not calcify in the absence of osteogenic medium. As a positive control, unsorted cells grown in OM for 6 days show the highest proliferation rate and moderate osteogenic differentiation. They have higher osteogenic differentiation potential than P2, P3 and P4, but lower than P1. It shows that unsorted cells are composed of heterogeneous cells with different osteogenic differentiation potential and different proliferation rate. The heterogeneous cell population may not accurately show the influence of biomaterials or growth factors on hMSC osteogenic differentiation. On the country, these sorted cells (P1–P3) can be used to study the effect of biomaterials or growth factors on hMSC osteogenic differentiation, and provide more reproducible results. In addition, as P1 cells are at late stage of osteogenic differentiation, our newly developed method could provide additional information about stem cell differentiation, such as the percentage of cells progressing into late stages of osteogenic differentiation (Fig. 2B).

In summary, a new method to observe mRNA expressions of two biomarker genes in individual live cells simultaneously, and isolate relatively homogeneous stromal cells based on mRNA expression has been developed. Using this method we could observe and quantify the mRNA expression in live cells, and analyze cell differentiation. The cells also can be sorted based on mRNA expression for further studies. In contrast to previous isolation methods which are limited by available surface markers, isolation of cells using mRNA specific probes provides the opportunity to investigate intracellular markers, such as transcription factors Runx2 or Sox9, and isolate relatively homogeneous cell populations. Adapting the transcription factors investigated will allow this method to be utilized for other cell phenotypes.

## Conclusion

We established a new method which offers an excellent non-destructive tool to analyze cell differentiation at the individual cell level, e.g. number of responding cells, as well as for whole population. Relatively homogenous and functionally similar cell populations can be isolated using this new method. By identifying and isolating differentiating cells at early time points, prospective analysis of differentiation is also possible, which could lead to a greater understanding of MSC differentiation.

## Additional Information

**How to cite this article**: Li, B. *et al*. Monitoring live human mesenchymal stromal cell differentiation and subsequent selection using fluorescent RNA-based probes. *Sci. Rep.* **6**, 26014; doi: 10.1038/srep26014 (2016).

## Supplementary Material

Supplementary Information
