# Corrigendum: Monitoring live human mesenchymal stromal cell differentiation and subsequent selection using fluorescent RNA-based probes

**DOI:** 10.1038/srep36429

**Published:** 2016-11-08

**Authors:** Bojun Li, Ursula Menzel, Claudia Loebel, Hagen Schmal, Mauro Alini, Martin J. Stoddart

Scientific Reports 6: Article number: 26014; 10.1038/srep26014 published online: 05 20 2016; updated: 11 08 2016.

This Article contains an error in the Introduction section,

“Gold nanoparticles are covalently labelled with capture oligonucleotides specific for Runx2 or Sox9 genes, and a fluorescently labelled short peptide. If complimentary mRNA is present, this peptide leaves the gold nanoparticle and begins to emit fluorescence”.

should read:

“Gold nanoparticles are covalently labelled with capture oligonucleotides specific for Runx2 or Sox9 genes, and a fluorescently labelled short oligonucleotide. If complimentary mRNA is present, this oligonucleotide leaves the gold nanoparticle and begins to emit fluorescence”.

