# Supplementary material for: Monitoring live human mesenchymal stromal cell differentiation and subsequent selection using fluorescent RNA-based probes
Source: Sci Rep. 2016 May 20;6:26014. doi: 10.1038/srep26014 (PMC4873741; doi:10.1038/srep26014)
Supplement: Supplementary Information [file srep26014-s1.pdf]

# Monitoring live human mesenchymal stromal cell differentiation and subsequent selection using fluorescent RNA-based probes


## Author list and affiliations:

Bojun Li[1], Ursula Menzel[1], Claudia Loebel[1], Hagen Schmal[2,3], Mauro Alini[1], Martin J. Stoddart[1,2]*


## Supplementary data:

Figure S1: Gating strategy for analysis of hBMSC by flow cytometry. A-C: DAPI negative (live) cells were selected (DAPI exclusion gate) (A) and then gated for the hBMSC population based on forward scatter (FSC) and side scatter (SSC) (B). Doublets were excluded applying a FSC-W vs FSC-A gate (C). This gating strategy was applied to all flow cytometry data presented in this manuscript.

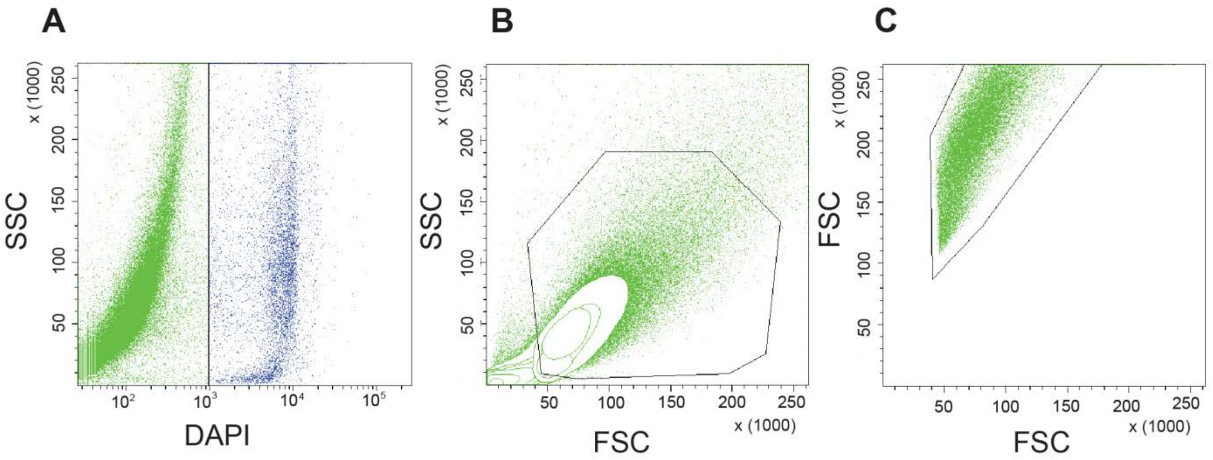

Figure S2: Fluorescence intensity of hBMSCs incubated simultaneously with Runx2-Cy3 and Sox9-Cy5 probes in GM or OM. For Runx2 80% cells in GM and 83% cells in OM took up SmartFlare™ Runx2-Cy3 probes and showed fluoresce signals (A). For Sox9 97% cells in GM and 90% cells in OM took up SmartFlare™ Sox9-Cy5 probes and showed fluorescence signals (B).

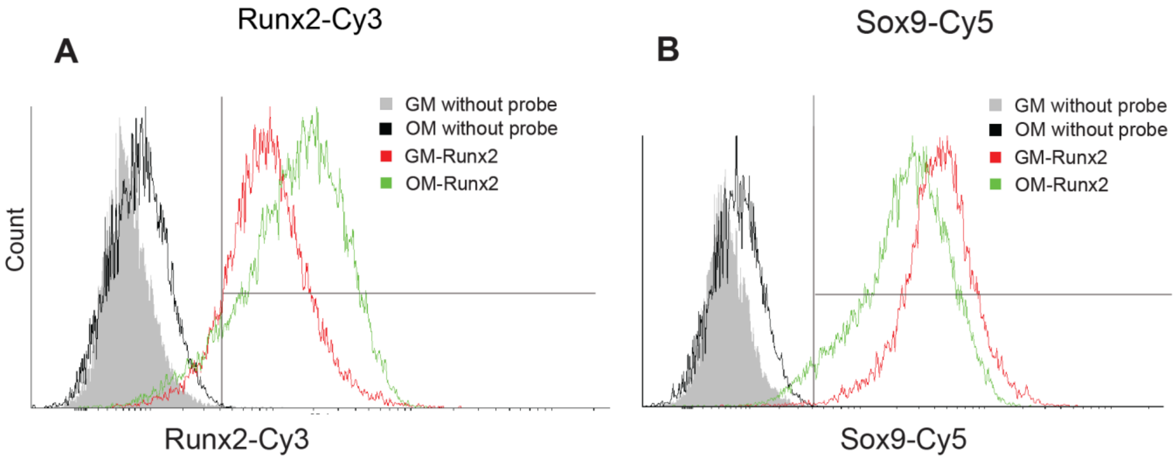

Figure S3: Real time PCR results for Collagen I, ALP and human Osteocalcin expression in sorted hBMSCs (P1-P4) based on fluorescence intensity for Runx2-Cy5 and Sox9-Cy3 expression and unsorted cells in GM or OM from 4 different donors. (GM: unsorted cells in GM, OM: unsorted cells P1: low Sox9/ low Runx2, P2: med Sox9/ med Runx2, P3: high Sox9/ high Runx2, P4: high Sox9/ med Runx2). mRNA expressions are normalized to unsorted cells in GM

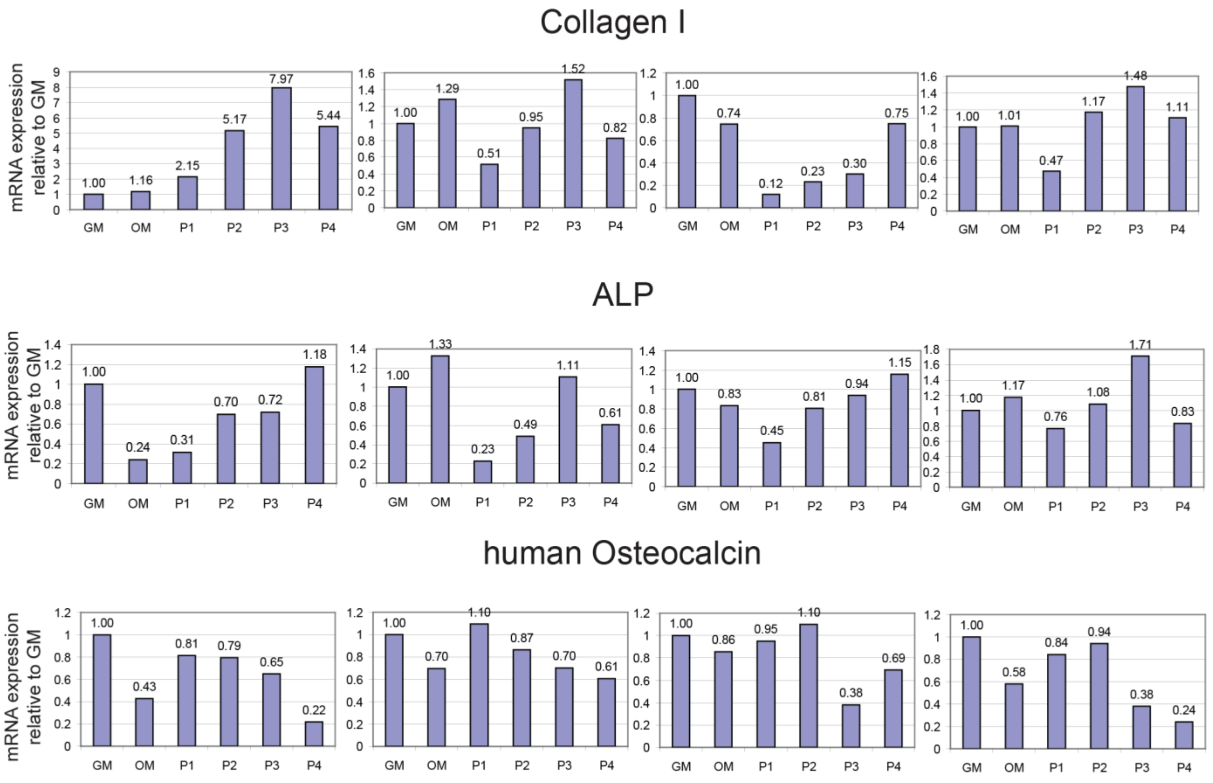


Figure S4: Alizarin red staining of sorted cells and unsorted cells (A,C), based on fluorescence intensity for Runx2-Cy5 and Sox9-Cy3 expression, from 2 donors after 3 week culture in GM or OM. Microscope view of Alizarin red staining of sorted cells and unsorted cells (B,D) Scale bar=100µm

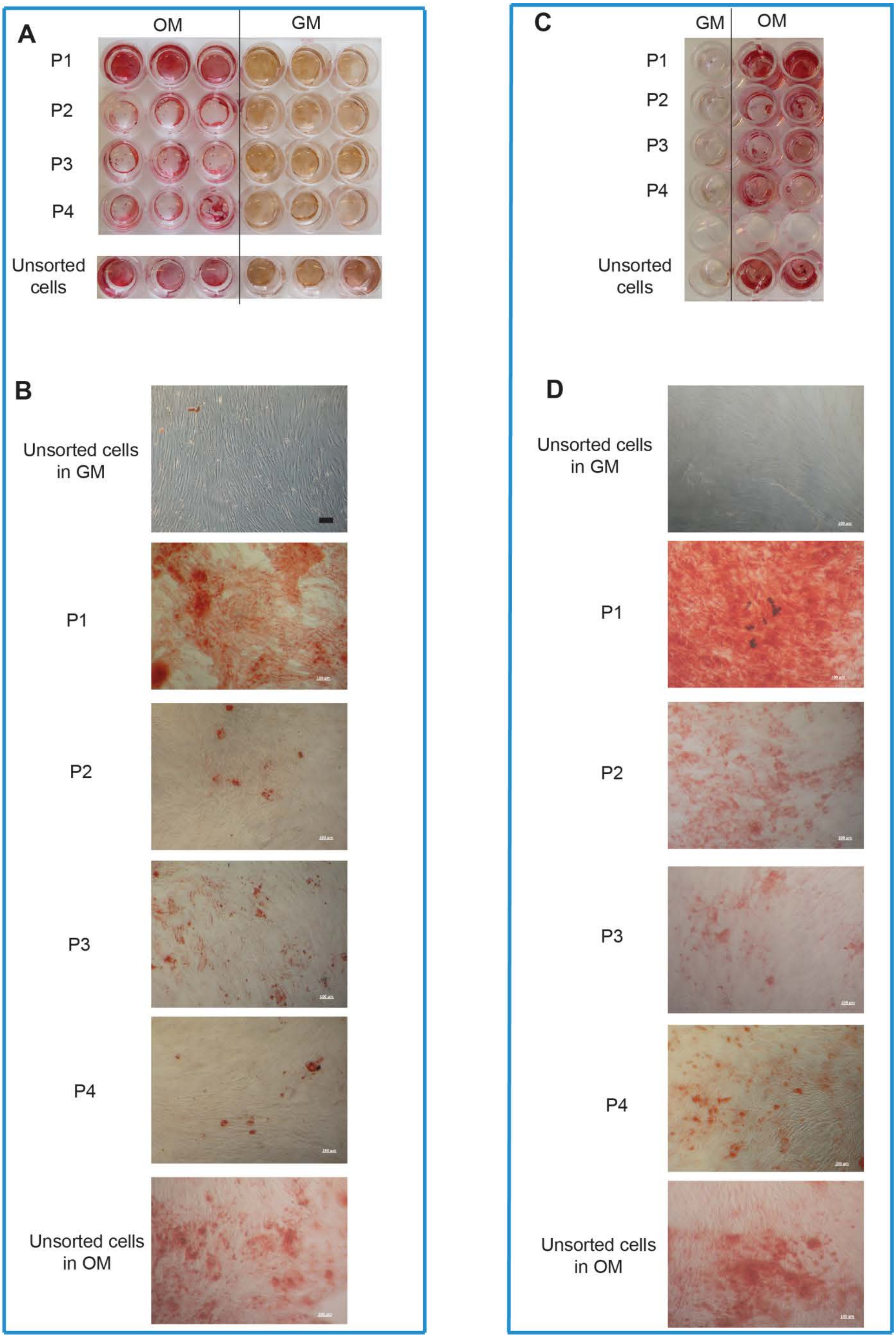
A
OM
GM
P1
P2
P3
P4
Unsorted cells
C
GM
OM
P1
P2
P3
P4
Unsorted cells
B
Unsorted cells in GM
P1
P2
P3
P4
Unsorted cells in OM
D
Unsorted cells in GM
P1
P2
P3
P4
Unsorted cells in OM
